# Treatment for Altered Passive Eruption: A 30-Month Follow-Up Case Study With a Digital Workflow Approach

**DOI:** 10.7759/cureus.82240

**Published:** 2025-04-14

**Authors:** Guenther Schuldt Filho, Christopher Basily, Arsalan Danesh, Márcio de Carvalho Formiga, Paulo G Warmling

**Affiliations:** 1 Periodontology, Nova Southeastern University, Clearwater, USA; 2 Dentistry, Nova Southeastern University, Clearwater, USA; 3 Oral Implantology, Univali São José, Florianopolis, BRA; 4 Operative Dentistry, Universidade do Sul de Santa Catarina (Unisul), Palhoça, BRA

**Keywords:** crown lengthening, dental esthetics, gingivectomy, periodontics, smile

## Abstract

Digital workflow has become an important tool for surgical dentistry and helps clinicians plan and treat several conditions. The excessive gingival display, also known as a gummy smile, is one of the conditions where digital planning and 3D-printed surgical guides bring benefits to both patients and surgeons, by increasing the accuracy of the execution, as close as possible, to the proposed planning. The objective of this case report is to show a case of altered passive eruption treated with aesthetic crown lengthening with the use of a 3D-printed surgical guide. After the patient accepted the proposed treatment plan, a surgical guide was printed according to the digital planning. The surgery had an initial gingivectomy guided by the 3D-printed guide, and after raising the mucoperiosteal flap, osteotomy was also guided by a 3D-printed guide. After three months, the patient was sent to the prosthodontist to replace a composite veneer on the left upper central incisor, with the margins exposed after the gingival healing. With 30 months of follow-up, the patient presents great margin stability and periodontal health. The use of digital workflow appears to facilitate the predictability of periodontal aesthetic treatments and may have more applicability shortly.

## Introduction

The demand for aesthetics is of paramount importance nowadays in the young population, especially due to the strong social media appeal, and to achieve it, a harmonious smile plays an important role [1]. A perfect smile is composed of some particular characteristics, such as good symmetry and well-aligned teeth, gingivae, and lips, with harmony with the facial composition. The architecture of the gingiva surrounding natural anterior teeth and how it is displayed during dynamic functional lip movements significantly influences the dento-gingiva aesthetics [2]. Excessive gingival display (EGD) is a very common condition that may interfere with the harmony of the face. Commonly known as a gummy smile, EGD is a multifactorial non-pathological condition defined as a high smile line that shows more than 2 mm of the gingiva when the patient is smiling, and is more prevalent in young and female subjects [3].

As one of the etiology factors of gummy smile, altered passive eruption (APE) is the most common dentoalveolar condition and can be classified into two distinct types (I and II) depending on the amount of keratinized tissue available. These two types can also be subdivided into subcategories (A and B), based on the relationship between the bone crest and the cementoenamel junction (CEJ) of the tooth [4]. EGD etiology may also include dentoalveolar or non-dentoalveolar conditions. One of the most common treatments for EGD due to APE is the aesthetic crown lengthening, which consists of gingivectomy with osteotomy and osteoplasty, in patients who do not feel comfortable with a gummy smile. The rationale of aesthetic crown lengthening to correct an APE is to keep 3 mm from the CEJ to the bone crest to provide space to re-establish the biologic width (e.g., the natural distance between the base of the gingival sulcus and the height of the alveolar bone) and at the same time expose the anatomical crown of the teeth. The concept of biologic width comes from a classic histologic study, measuring the average dimension of the epithelial junction (0.97 mm) and connective tissue attachment (1.07 mm) in humans [5]. The integrity of these structures is considered a necessary step in restorative and prosthetic rehabilitations to obtain and maintain healthy soft tissues. We must keep in mind that aesthetic crown lengthening to treat APE is usually done in the buccal side of maxillary anterior teeth, and does not involve papillae resection, compared to the usual crown lengthening, especially in cases where any restorative treatment will be performed after surgery [6].

The suggestion of employing a digital process for managing patients with APE has been put forth in cases involving both restorative treatments and situations where no restorative treatment is expected. The digital workflow has been improved in recent years, allowing meticulous treatment planning facilitated by digital tools, minimizing the risks of incorrect incision placement and excessive or insufficient bone removal, besides maximizing the accuracy of the osteotomy [7]. The integration of digital planning into the treatment of APE allows for a more precise treatment, as the provider can visualize the expected outcome, make well-informed decisions, and carry out crown lengthening procedures with heightened accuracy, leading to a faster surgery. A cone-beam computed tomography (CBCT) scan is used to analyze the CEJ and its relationship to the level of the alveolar bone crest. CBCT-derived DICOM (Digital Imaging and Communications in Medicine) files undergo a conversion process to STL (Standard Tessellation Language) format. Subsequently, these STL files are overlaid with others obtained from intraoral scans. The virtual design of the surgical guide is then created. Data are then transferred to a 3D printer, where the guide is then printed [8].

Therefore, the present article aims to report a clinical case of EGD attributed to APE treated with aesthetic crown lengthening guided by a 3D-printed double guide and the posterior prosthetic rehabilitation of a single ceramic veneer on the central incisor. This study has a sample size of one. In future studies, the efficacy of this technique needs to be assessed to determine if it is truly better than the non-surgical guided method.

## Case presentation

**Figure 1:**
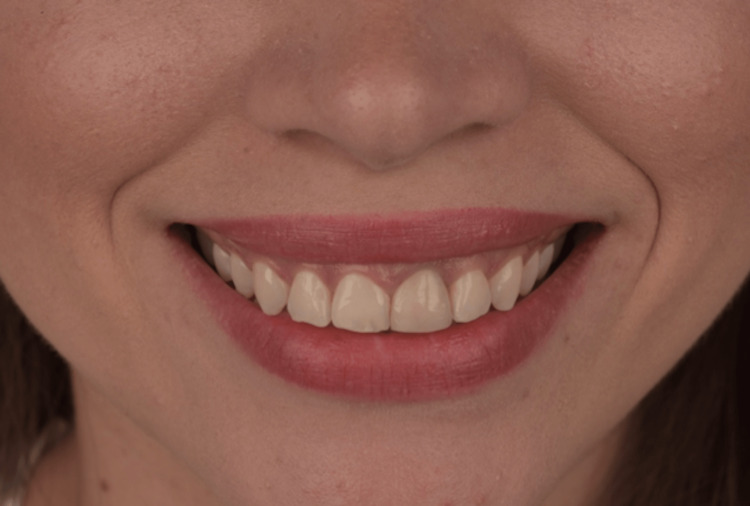
Initial extra oral view of the patient’s smile.

**Figure 2:**
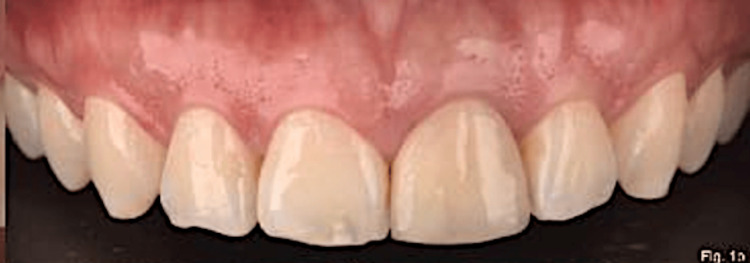
Initial buccal view of the maxillary anterior teeth.

**Figure 3:**
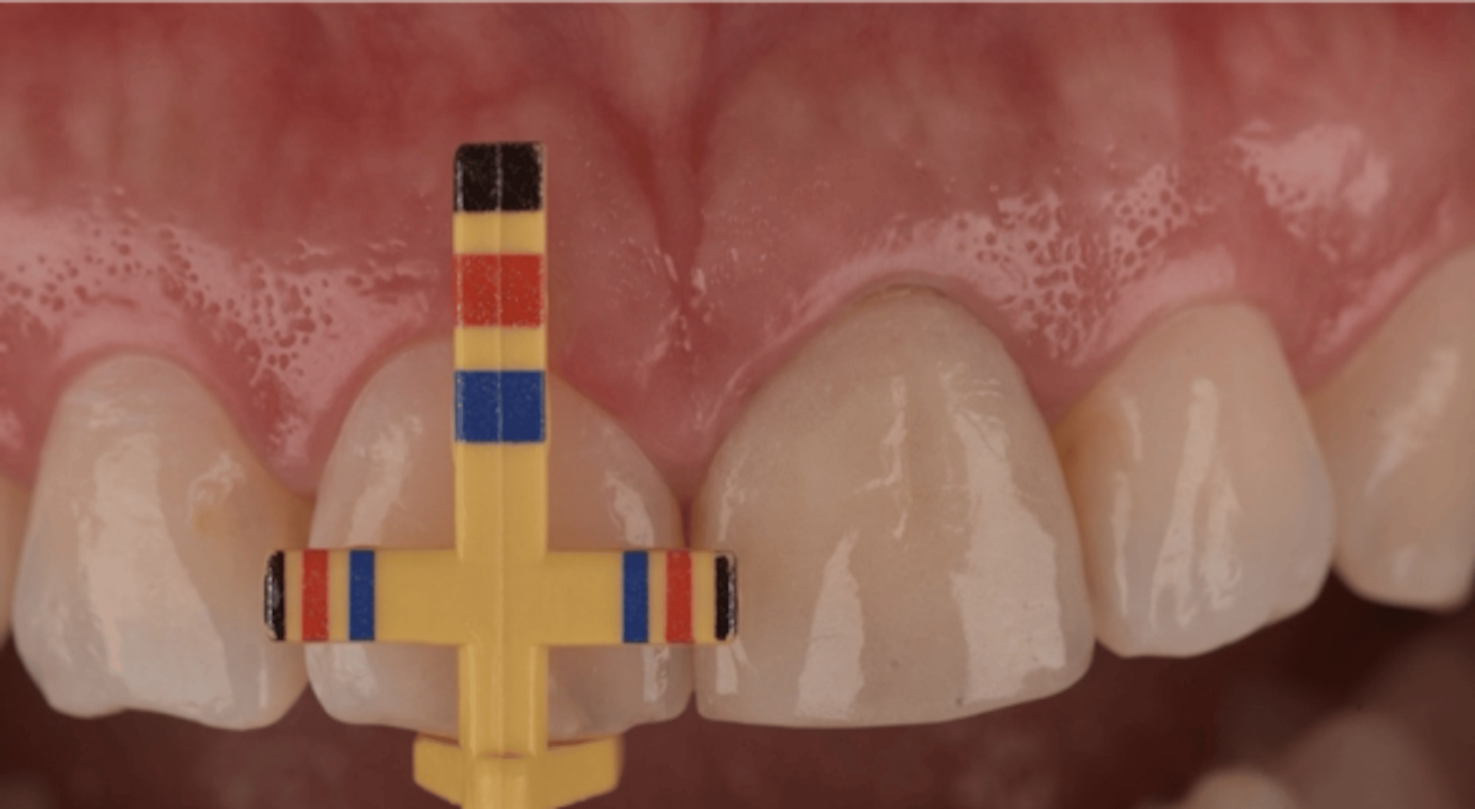
Clinical examination using a Chu proportion gauge.

**Figure 4:**
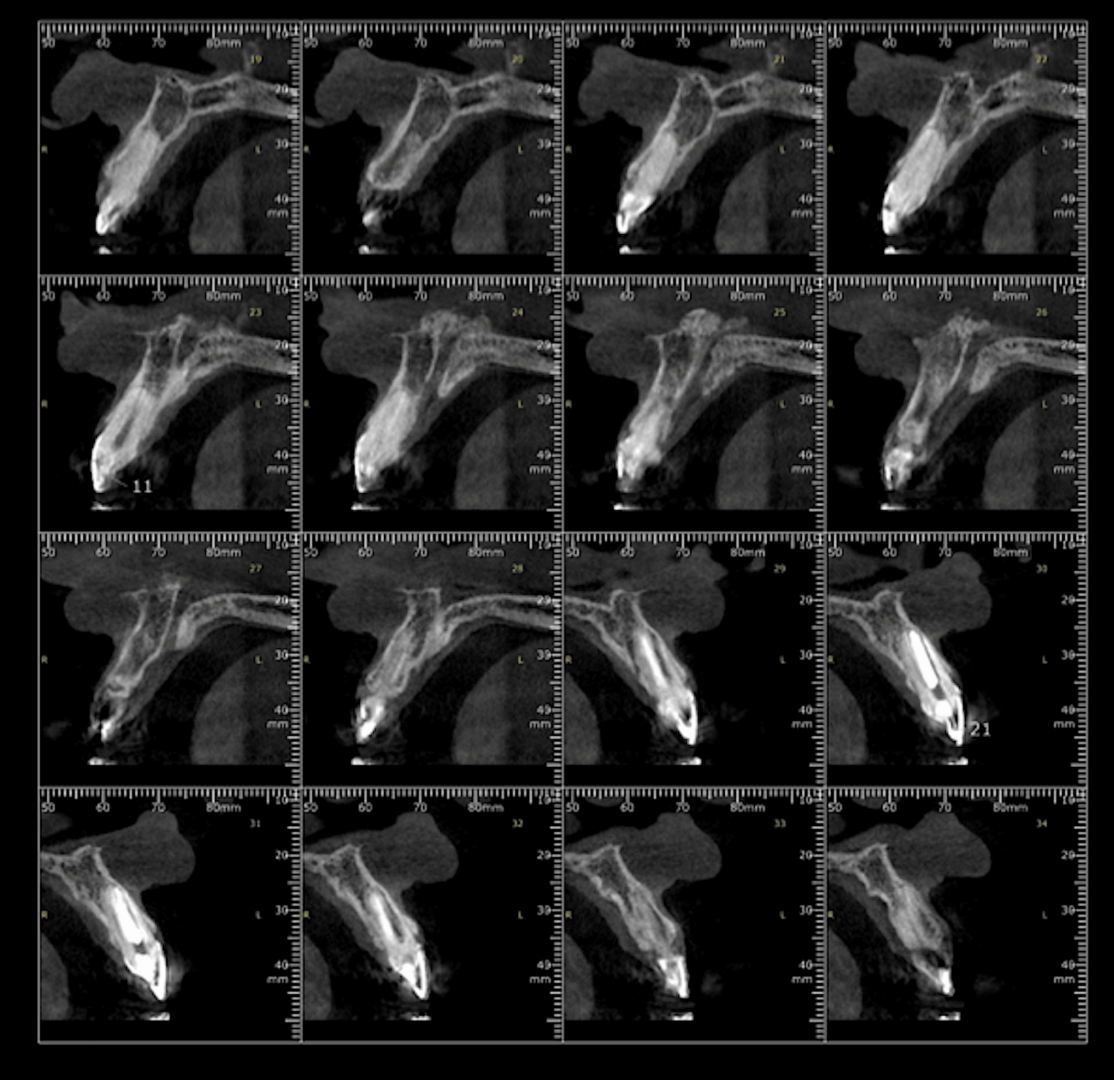
Cone-beam computed tomography analysis of the upper anterior sextant.

A 31-year-old woman with apparent good health presented to our private practice with the complaint of dissatisfaction with her smile. When evaluating intra and extra orally, we were able to verify that she had a gummy smile (Figure 1), short anterior teeth (confirmed by the Chu aesthetic gauge) (Figures 2, 3), and an aesthetically satisfactory veneer on the left maxillary central incisor, with a dark color in the gingiva, suggesting a dark root, and interfering in the aesthetic result. No signs of any periodontal problems were diagnosed, and no bleeding on probe was noted. Preliminary to treatment, a comprehensive clinical and radiographic examination was conducted. CBCT was done to analyze the distance between the bone crest and the CEJ. It was observed that the bone crest closely followed the CEJ, indicating a precise need for bone resection (Figure 4). On the clinical examination, we stated a broad band of keratinized gingiva (KG), classifying the patient under type 1 - subtype B9. Our proposed treatment plan involved a gingivectomy combined with osseous resection, guided by a 3D-printed surgical guide, and the replacement of the veneer restoration on the left maxillary central incisor after the healing period. The patient approved our treatment suggestion and signed an informed consent prior to the beginning of the treatment.

**Figure 5:**
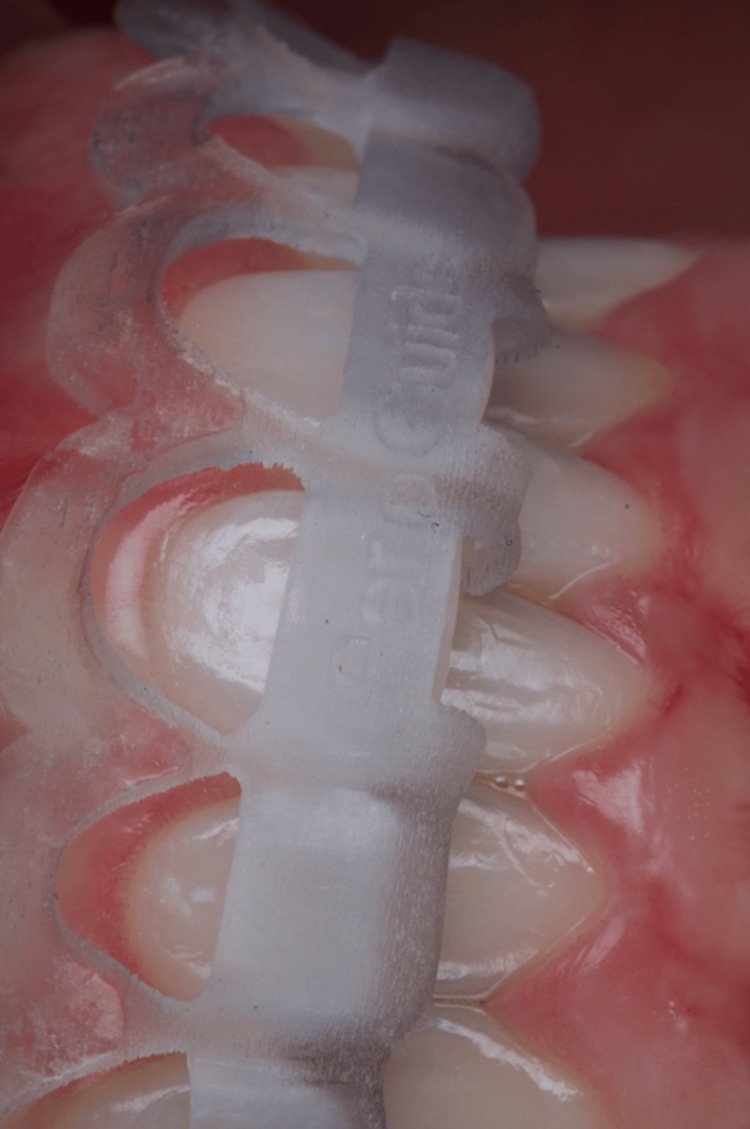
3D-printed guide positioned on teeth.

**Figure 6:**
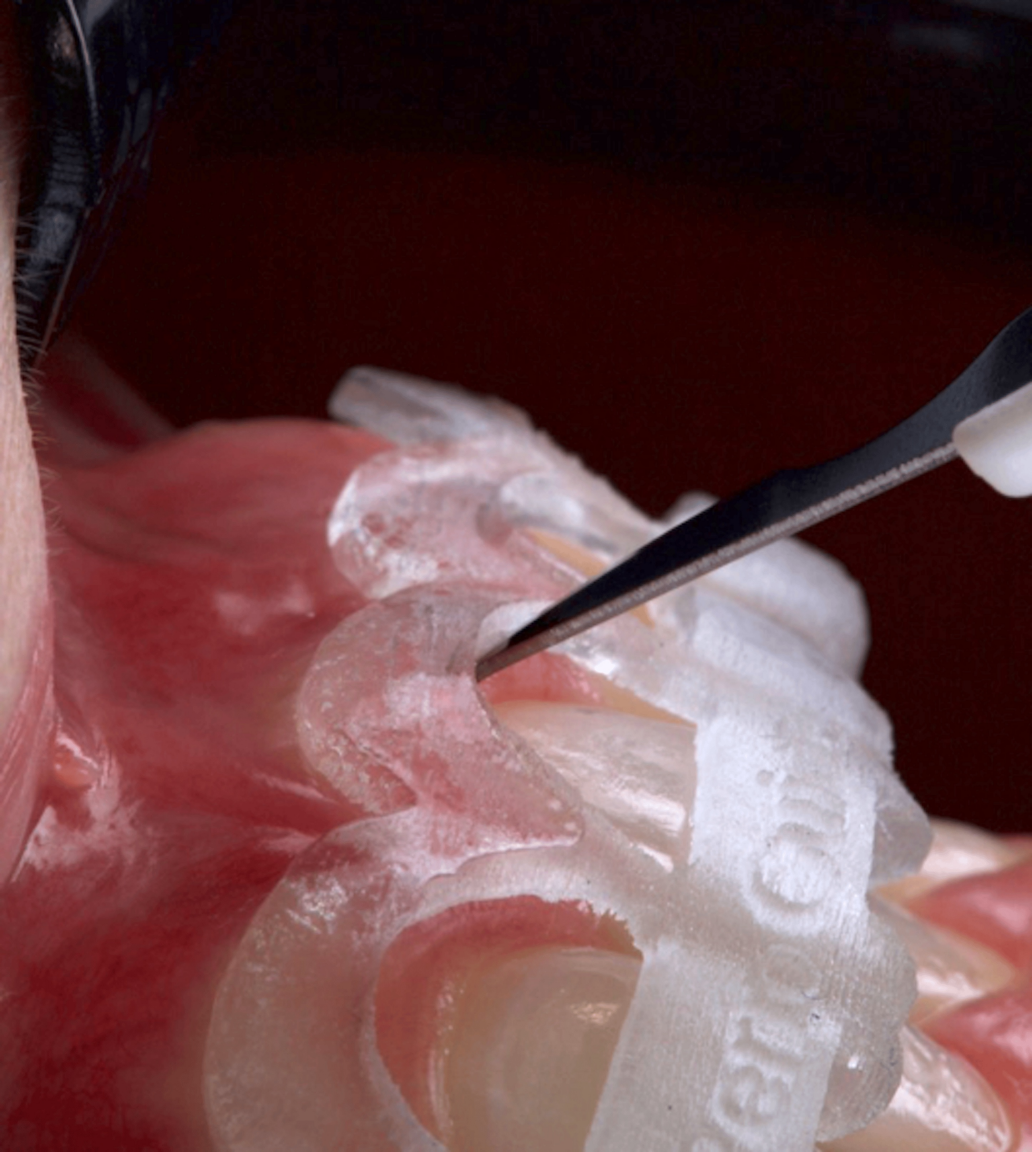
Close detail of the 15c blade performing the gingivectomy.

**Figure 7:**
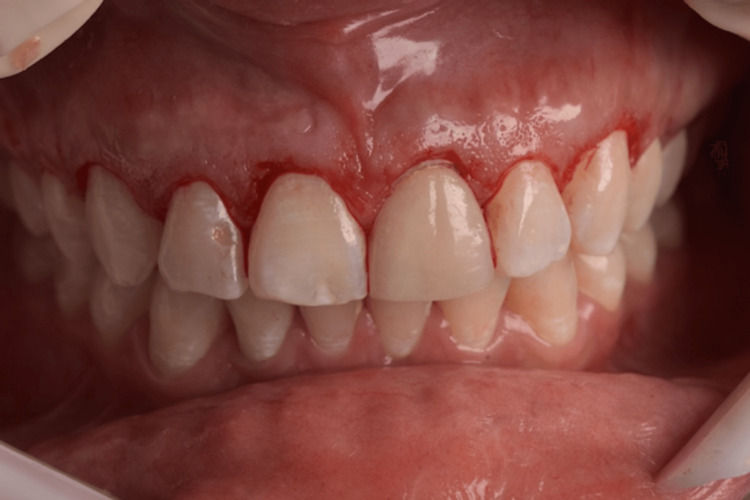
Buccal view of the new gingival margins post gingivectomy.

**Figure 8:**
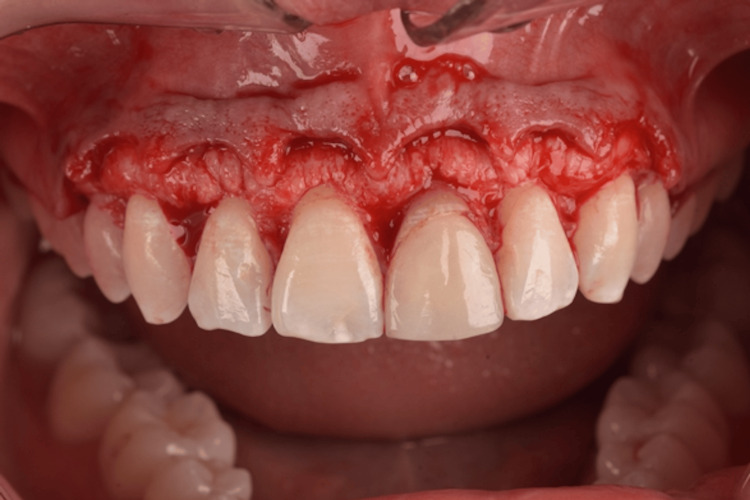
Buccal view of the surrounding bone after raising the full thickness flap.

**Figure 9:**
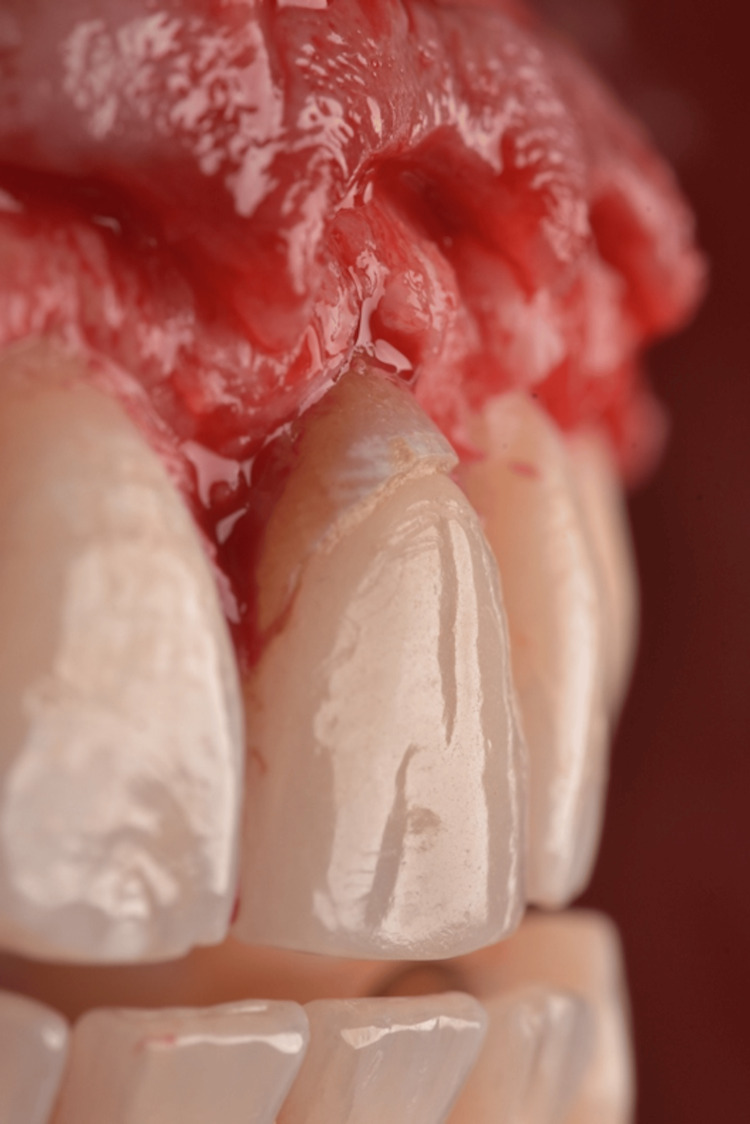
Close view of the appearance of the bony architecture and presence of shallow osseous ledges before osteotomy, and also the lack of marginal adaptation of the old ceramic veneer.

The surgical guide for the aesthetic crown lengthening was designed to establish the relationship between the CEJ and the alveolar crest. The resulting surgical guide was produced using a 3D printer, featuring key windows on the buccal aspects of the teeth to guarantee proper seating during surgery (Figure 5). Two parallel scalloped lines delineate the incision line for the coronal gingivectomy procedure and the line for osseous resection. The patient was medicated with 2 g of amoxicillin one hour before the procedure. Extraoral asepsis was performed with a 0.2% chlorhexidine digluconate solution, and then the patient used a mouthwash solution with 0.12% chlorhexidine digluconate for one minute. After that, infiltrative local anesthesia with 4% articaine (Nova DFL, Rio de Janeiro, Brazil) was administered. The primary internal bevel incision was performed with a micro blade (Spoon Blade SB002-MJK, São Paulo, Brazil), with intrasulcular incisions (Figure 6) from the right premolar to the left premolar. Next, a 15C blade (Swann-Morton, England) was used to contour the internal window of the surgical guide, delimitating the height for collar removal (Figure 7), and then raising a full-thickness flap to expose the crestal bone to be removed (Figure 8). In some of the teeth, we could see the crestal bone above the CEJ junction, confirming the CBCT images. The lack of marginal adaptation of the ceramic veneer to be changed also became evident. Osteoplasty was performed using chisels, aiming to remove non-supporting bone and to eliminate any osseous ledges. The buccal ostectomy was then performed using a high-speed spherical diamond bur under copious irrigation, using the surgical stent as a guideline for the amount of bone removal. Vertical mattress sutures with 5.0 nylon monofilament were used to approximate partially raised interdental papillae (Figure 9). The patient was medicated for pain and edema control with paracetamol 750 mg and ibuprofen 600 mg every eight hours for three days, and was recommended to put ice bags on the region for 10 to 15 minutes every hour for 48 hours. Also, the patient was advised to eat only soft and cold food for three days.

**Figure 10:**
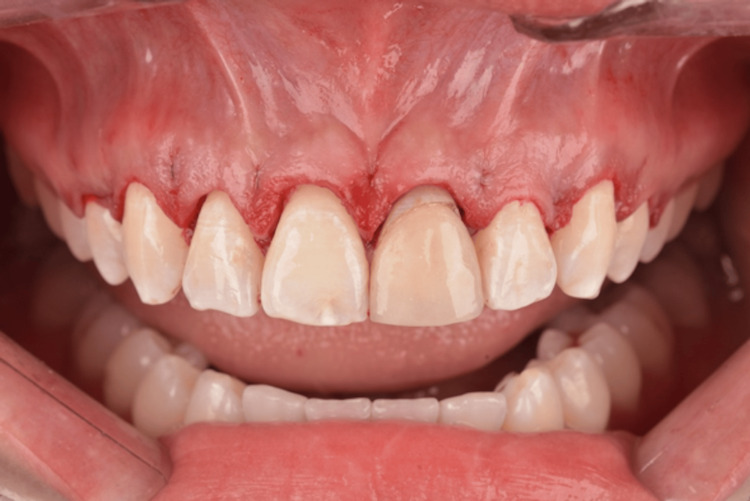
Buccal view immediately after interdental papillae vertical mattress sutures.

**Figure 11:**
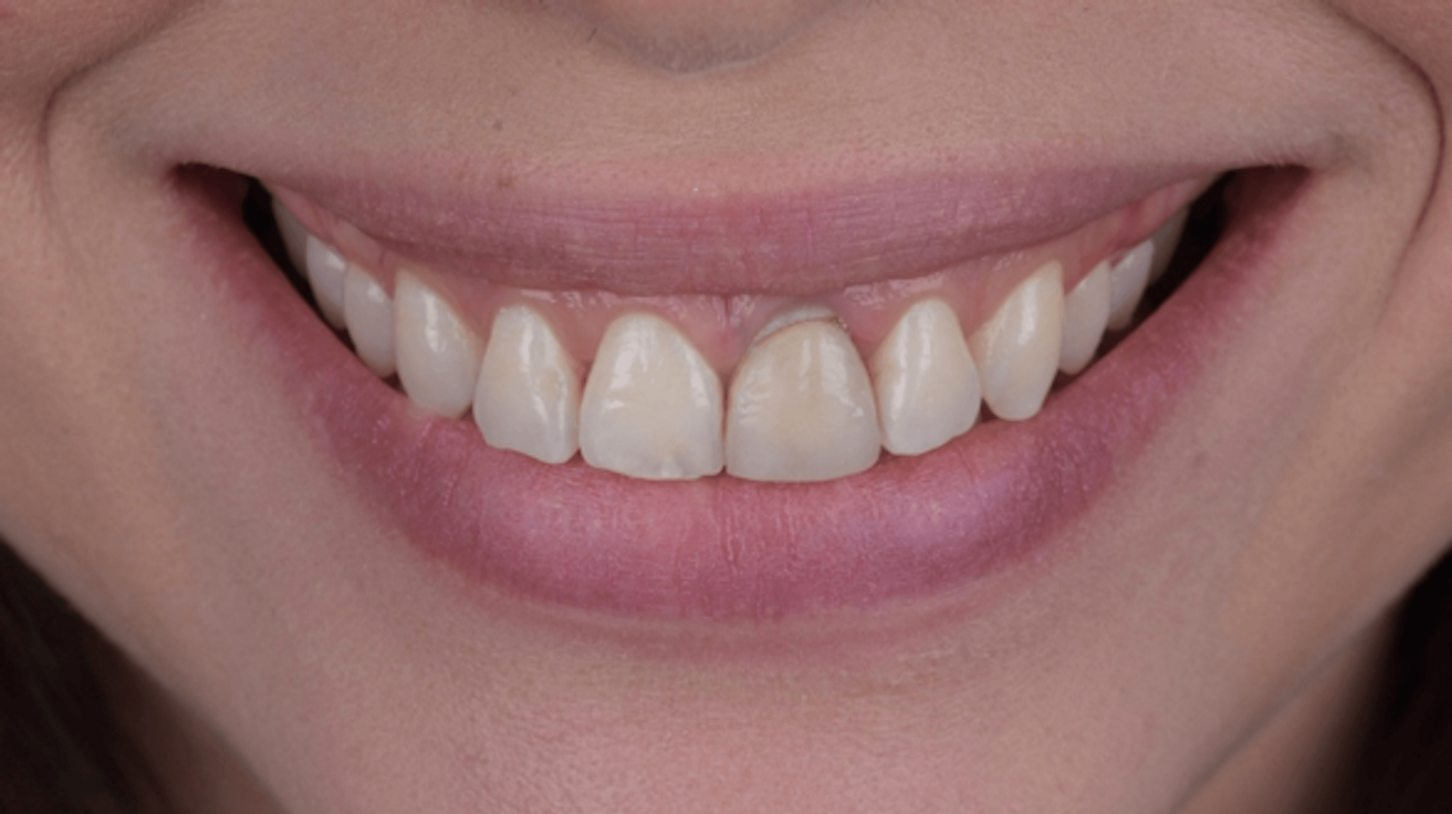
Extra oral front view of the patient’s smile 90 days after surgery.

**Figure 12:**
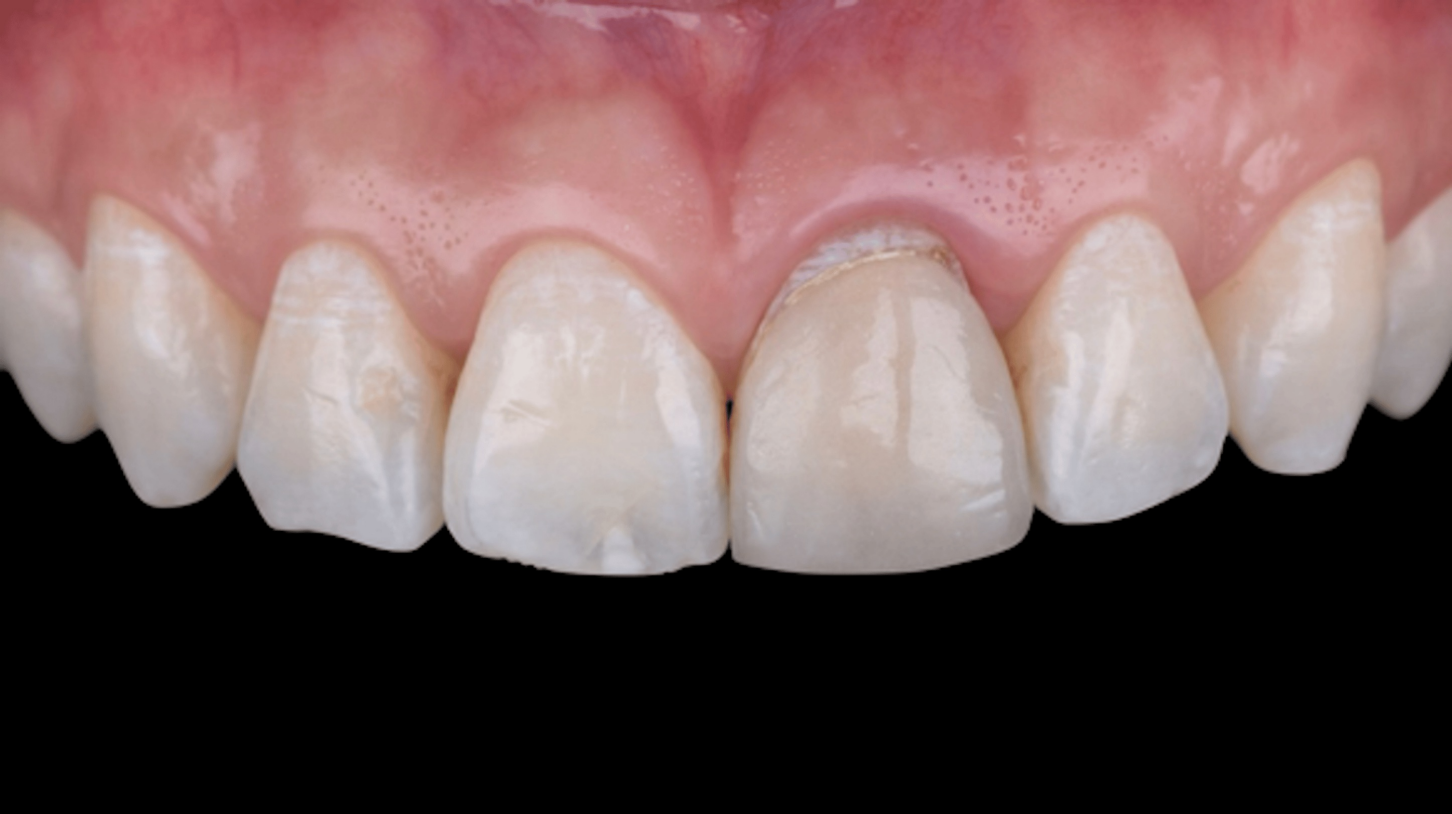
Intra oral buccal view 90 days after surgery.

**Figure 13:**
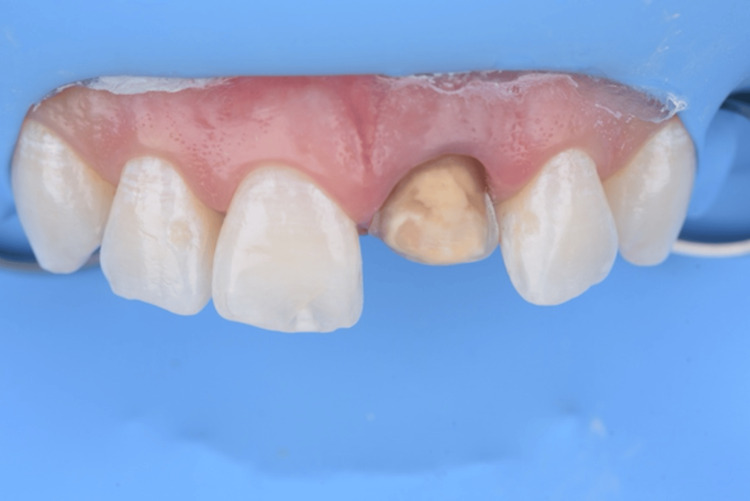
Buccal view of the bleached teeth with adapted absolute isolation prior to cementing the new ceramic veneer.

**Figure 14:**
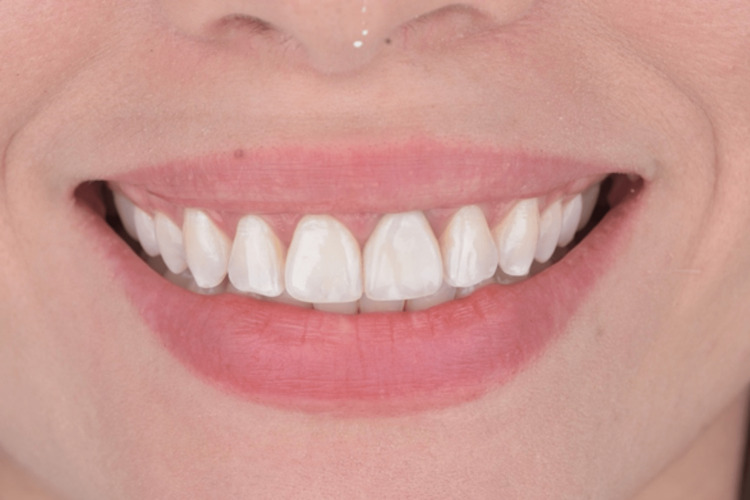
Extra oral front view of the new ceramic veneer cemented.

**Figure 15:**
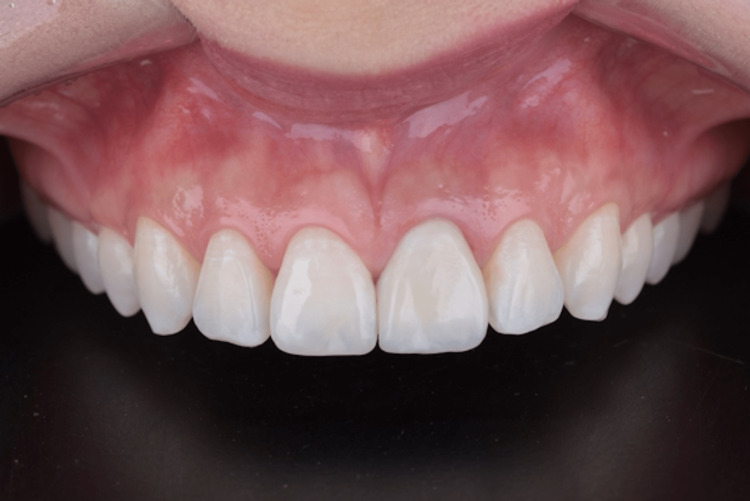
Intra-oral buccal view 30 months after surgery.

After 90 days of healing, the patient returned for a postoperative control appointment, with the margin of the ceramic veneer exposed, as expected, but with an evident reduction of the gummy smile (Figure 10). On the intraoral examination, periodontal health was noted, with no more than 3 mm on probing, so bleeding on probe, with good gingival contour and color was noted (Figure 11). The patient was sent to the prosthodontist, where she had their teeth bleached, and only after that, the left upper central incisor was prepared to receive a new indirect ceramic veneer (Figures 12-14). It is important to mention that tooth preparation and tooth bleaching were only performed after visual and clinical analysis that the soft tissues were completely healed. Thirty months after surgery, the patient returned for a biofilm control appointment, where the maintenance of the good result of the surgical and prosthetic treatments was highlighted (Figure 15).

## Discussion

Gingivectomy with crown lengthening with osteotomy is one of the most commonly used procedures to treat EGD in patients in disagreement with their gummy smile. In some cases, the treatment plan may include apically repositioned flap, the use of botulinum toxin and myotomy of lip-elevating muscles [9,10], or the use of a polymethylmethacrylate (PMMA) cement to fill the subnasal depression, and with that, reduce the lip movement and avoid the upper lip to lie on it, and diminish the gingival exposure [11]. Other alternatives to perform the osteotomy without rotatory instruments, or even with a flapless approach, have been studied, showing good results, and with no differences in the clinical outcomes compared to the conventional technique [12]. In our case report, the aesthetic crown lengthening with an open flap to reduce the bone volume was the elected technique, and was able to solve the patient’s complaint of gummy smile, avoiding the need for any other procedure.

The amount of marginal gingiva to be removed in this case was determined clinically by photographs and the Chu proportion gauge [13], among of course, the periodontist experience, and confirmed by the digital documentation and planning that allows a meticulous planning of osseous resection and gingivectomy amounts before the actual surgical appointment, so higher efficiency and precision during the surgical procedure are ensured. A 3D printing surgical guide was used to assess the ideal amount of soft and hard tissue reduction and to diminish the possibility of errors. The use of digital workflow-based guides requires increased treatment planning time and additional costs, but the popularity of such modality is increasing for periodontal procedures [8]. Digital treatment planning is based on diagnostic methods such as CBCT for the analysis of hard and soft tissues and may improve the communication between surgeon and restorative dentist, and with that, increase the accuracy and predictability of surgical procedures, achieving better aesthetic and biological outcomes in patients with EGD [12].

The patient also needed to replace an old veneer, so we waited three months for gingival margin stability before initiating the prosthetic management of the aforementioned tooth [14]. The amount of osteotomy was digitally planned to maintain 3 mm of biological width between the margin of the restoration and the crestal bone after the procedure. This healing period and this 3 mm distance are preconized by other authors before any kind of restorative procedures, to assure there is no rebound of the margin, or recession as well. Tissue rebound, gingival recession, uneven margins, and loss of interdental papillae are among the most common complications in the aesthetic crown lengthening procedures [15], and may occur even in cases digitally planned, as ours. At the 30-month follow-up, we could state the stability of the gingival margins in this clinical case.

## Conclusions

Within the limitations of this case, we can conclude that the use of digital workflow in the aesthetic treatment of APE helped in the predictability of the final result of the case, guiding the surgeon based on the final prosthetic design. The digital workflow facilitated the diagnostic and surgical phases, providing more accuracy and predictability to the results. We recommend more long-term studies to assure the tissue stability of these cases.
